# Adipocyte-Specific Hypoxia-Inducible Factor 2α Deficiency Exacerbates Obesity-Induced Brown Adipose Tissue Dysfunction and Metabolic Dysregulation

**DOI:** 10.1128/MCB.00430-15

**Published:** 2016-01-19

**Authors:** Rubén García-Martín, Vasileia I. Alexaki, Nan Qin, María F. Rubín de Celis, Matina Economopoulou, Athanasios Ziogas, Bettina Gercken, Klara Kotlabova, Julia Phieler, Monika Ehrhart-Bornstein, Stefan R. Bornstein, Graeme Eisenhofer, Georg Breier, Matthias Blüher, Jochen Hampe, Ali El-Armouche, Antonios Chatzigeorgiou, Kyoung-Jin Chung, Triantafyllos Chavakis

**Affiliations:** a Department of Clinical Pathobiochemistry, Medical Faculty, Technische Universität Dresden, Dresden, Germany; b Institute of Clinical Chemistry and Laboratory Medicine, Medical Faculty, Technische Universität Dresden, Dresden, Germany; c Department of Medicine III, Medical Faculty, Technische Universität Dresden, Dresden, Germany; d Department of Ophthalmology, Medical Faculty, Technische Universität Dresden, Dresden, Germany; e Department of Psychiatry, Medical Faculty, Technische Universität Dresden, Dresden, Germany; f Department of Medicine I, Medical Faculty, Technische Universität Dresden, Dresden, Germany; g Department of Pharmacology and Toxicology, Medical Faculty, Technische Universität Dresden, Dresden, Germany; h Department of Endocrinology and Nephrology, University of Leipzig, Leipzig, Germany; i Center for Regenerative Therapies Dresden, Dresden, Germany; j Paul Langerhans Institute Dresden of the Helmholtz Center Munich at University Hospital and Faculty of Medicine, TU Dresden, Dresden, and German Center for Diabetes Research (DZD e.V.), Neuherberg, Germany

## Abstract

Angiogenesis is a central regulator for white (WAT) and brown (BAT) adipose tissue adaptation in the course of obesity. Here we show that deletion of hypoxia-inducible factor 2α (HIF2α) in adipocytes (by using Fabp4-Cre transgenic mice) but not in myeloid or endothelial cells negatively impacted WAT angiogenesis and promoted WAT inflammation, WAT dysfunction, hepatosteatosis, and systemic insulin resistance in obesity. Importantly, adipocyte HIF2α regulated vascular endothelial growth factor (VEGF) expression and angiogenesis of obese BAT as well as its thermogenic function. Consistently, obese adipocyte-specific HIF2α-deficient mice displayed BAT dysregulation, associated with reduced levels of uncoupling protein 1 (UCP1) and a dysfunctional thermogenic response to cold exposure. VEGF administration reversed WAT and BAT inflammation and BAT dysfunction in adipocyte HIF2α-deficient mice. Together, our findings show that adipocyte HIF2α is protective against maladaptation to obesity and metabolic dysregulation by promoting angiogenesis in both WAT and BAT and by counteracting obesity-mediated BAT dysfunction.

## INTRODUCTION

The hypoxia response is mediated by the heterodimeric hypoxia-inducible factors (HIFs) comprising an α subunit (HIF1α or HIF2α) that is regulated by oxygen and an oxygen-insensitive β subunit. Hypoxia prevents hydroxylation of HIFα subunits by prolyl hydroxylases (PHDs), thereby leading to inhibition of degradation of the HIFα subunits, their stabilization, and the subsequent HIF-dependent upregulation of genes essential for cellular adaptation to and cell survival under hypoxic conditions (reviewed in reference 1).

In obesity, excessive lipid storage and adipocyte hypertrophy are thought to result in hypoxia in white adipose tissue (WAT) and brown adipose tissue (BAT) (2–6), although there is some controversy concerning oxygen levels in obese AT (7). The presence of hypoxia in WAT may be associated with increased inflammation (2, 4, 5); hypoxia also triggers an angiogenic response, including upregulation of the major angiogenic factor, vascular endothelial growth factor (VEGF), in adipocytes (4). The angiogenic response is crucial for the adaptation of the WAT in obesity. Previous studies have demonstrated that defective angiogenesis in obese WAT may promote insulin resistance, inflammation, and adipocyte apoptosis (3, 8, 9). On the other hand, mice with adipocyte overexpression of VEGF family member A (VEGF-A) are protected against the adverse effects of a high-fat diet (HFD) (8, 10, 11). In obese WAT, enhanced expression of both HIF1α and HIF2α is observed (2, 6, 12). Previous studies have functionally implicated HIF1α (6, 12–14) and different PHDs (15, 16) in the process of obesity. In contrast, less is known about the role of HIF2α. HIF2α heterozygous null mice were recently shown to have reduced insulin sensitivity and enhanced AT inflammation upon HFD (17) and HIF2α was found to regulate lipid metabolism in hepatocytes (18–20), whereas fewer data exist on the specific role of HIF2α in adipocytes (6). This is particularly important given several findings demonstrating that regulation of VEGF-A and angiogenesis in WAT is not dependent on HIF1α. Whereas adipocytes lacking HIF1β (the common and obligate partner for HIF1α and HIF2α) showed reduced VEGF expression (21), transgenic mice overexpressing HIF1α in adipocytes did not show any upregulation in VEGF-A expression or other proangiogenic factors (12). In addition, mice lacking adipocyte HIF1α showed no vascular alterations compared to HIF1α-proficient mice (6). These observations point to a potential role of HIF2α in the WAT for the adaptive response to obesity that remains to be established.

BAT is also a highly vascularized tissue; the grade of vascularization determines its ability for lipid consumption and its thermogenic function (3, 22). Increased BAT activity results in improved insulin sensitivity and glucose homeostasis (reviewed in reference 23). Norepinephrine derived from sympathetic nerves is a central player in inducing expression of both VEGF and the major thermogenic factor uncoupling protein 1 (UCP1) (24). Catecholamine-mediated induction of UCP1 requires a signaling cascade involving cyclic AMP (cAMP), protein kinase A, and PGC1α (reviewed in reference 23). VEGF resulting from beta adrenergic receptor stimulation promotes BAT angiogenesis and functionality (3, 8, 10, 11, 25–27). Interestingly, UCP1-deficient mice display angiogenesis despite the absence of hypoxia in their BAT (25, 28, 29); thus, hypoxia in BAT is not an absolute prerequisite for stimulation of angiogenesis. Hypoxia may collaborate with norepinephrine in upregulating VEGF expression in brown adipocytes (24), whereas activation of HIF1α in these cells may occur even without hypoxia (28). However, HIF1α does not regulate expression of VEGF (3) or UCP1 (6) in BAT. On the other hand, although HIF2α expression is induced by cold exposure (25), its potential role in the adaptive response of BAT to obesity and cold exposure has not been addressed thus far.

To address the aforementioned issues pertinent to the role of HIF2α in both WAT and BAT, we generated mice with adipocyte-specific HIF2α deletion. We found that the lack of HIF2α in adipocytes resulted in systemic insulin resistance associated with reduced vascularization and a proinflammatory phenotype in both WAT and BAT in the course of obesity. In contrast, myeloid or endothelial HIF2α did not affect obesity-related metabolic dysregulation. In addition to reduced angiogenesis in the BAT, adipocyte HIF2α deficiency was associated with reduced expression of the major thermogenic factor UCP1 in obese BAT. Treatment with VEGF reversed WAT and BAT inflammation and BAT dysfunction in obese mice lacking adipocyte HIF2α, suggesting that the metabolic dysregulation observed in adipocyte HIF2α deficiency was, at least in part, mediated by diminished VEGF production. Thus, adipocyte HIF2α was identified as a factor contributing to the metabolic adaptation to diet-induced obesity in both WAT and BAT.

## MATERIALS AND METHODS

### Mice.

Mice carrying a floxed HIF2α (*Epas1*) allele (Jackson Laboratories, Bar Harbor, ME) (HIF2α^fl/fl^) were bred with mice carrying Cre recombinase under the control of Fabp4 promoter (Jackson Laboratories) to generate adipocyte-specific HIF2α knockout (KO) mice (AdHIF2KO) (Fabp4-Cre^+^ *Epas1*^fl/fl^). Similarly, *Epas1*^fl/fl^ mice were crossed with LysM-Cre mice (30) (Jackson Laboratories) to generate myeloid cell-specific HIF2α KO mice (MyeHIF2KO) (LysM-Cre^+^ *Epas1*^fl/fl^). Fabp4-Cre^−^ *Epas1*^fl/fl^ and LysM-Cre^−^ *Epas1*^fl/fl^ littermates were used as controls. For the study of endothelial HIF2α, we engaged a tamoxifen-mediated inducible deletion by using CreERT, whose expression is driven by stem cell leukemia promoter (Scl)-5′, which is specifically expressed in endothelial cells (31, 32). Scl-CreERT^+^ *Epas1*^fl/fl^ (EndHIF2KO) and Scl-CreERT^−^ *Epas1*^fl/fl^ littermate control mice received intraperitoneal (i.p.) tamoxifen (Sigma-Aldrich, Munich, Germany) (2 mg/mouse/day) at the age of 8 weeks. Eight- to 10-week-old male mice were fed a normal diet (ND) or high-fat diet (HFD) with 10% kilocalories from fat or 60% kilocalories from fat, respectively (Research Diets, New Brunswick, NJ), and feedings were conducted for up to 24 weeks. Animal experiments were approved by Landesdirektion Sachsen, Germany.

### *In vivo* metabolic analyses.

For the glucose tolerance test, mice were fasted overnight before intraperitoneal injection of d-(+)-glucose (Sigma-Aldrich, Munich, Germany) (1 g/kg of body weight). At the desired times, blood was collected via tail vein for measuring glucose levels with an Accu-Chek glucose meter (Roche, Mannheim, Germany). For the insulin tolerance test, mice were fasted 6 h before intraperitoneal injection of insulin (Lilly, Bad Homburg, Germany) (1 U/kg of body weight) and blood glucose levels were measured at the desired times. For glucose-stimulated insulin secretion, mice were fasted overnight before intraperitoneal injection of d-(+)-glucose (Sigma-Aldrich) (1 g/kg of body weight). At the desired times, blood was collected via tail vein for measuring plasma insulin with an enzyme-linked immunosorbent assay (ELISA) kit (Chrystal Chem, Cologne, Germany). Blood triglycerides and cholesterol were determined using an Accutrend Plus system (Roche). For analysis of fasted plasma samples, mice were fasted overnight (16 to 18 h), blood was collected via tail vein, and plasma leptin, adiponectin, insulin, and FGF21 were determined using ELISA kits (R&D Systems, Wiesbaden-Nordenstadt, Germany; Chrystal Chem) by following the manufacturer's instructions. For the lipid tolerance test, mice were fasted overnight before oral gavage of olive oil (Sigma-Aldrich) (5 μl/g of body weight) and blood triglycerides were determined as described above. For the determination of free fatty acids (FFAs), serum from mice fasted for 16 to 18 h was collected and a free fatty acid fluorometric assay kit was used (Cayman Chemical, Ann Arbor, MI). For *in vivo* insulin signaling pathway analysis, mice were fasted for 6 h before intraperitoneal injection of insulin (Lilly) (5 U/kg); 8 min thereafter, mice were euthanized and tissues were harvested and snap-frozen for further analysis. Lean and fat mass was measured in mice fed an HFD for 22 weeks by using computed tomography (CT) (33) (Skyscan 1178; Bruker, Rheinstetten, Germany). For cold-exposure experiments, obese mice fed for at least 19 weeks with an HFD were placed at 4°C overnight. Body temperature was measured at the desired times with a thermometer (Bioseb, Vitrolles, France). Thereafter, mice were sacrificed and tissues isolated and processed at 4°C.

### VEGF administration.

Controlled administration of VEGF was achieved via subcutaneous implantation of mini-osmotic pumps (Alzet, CA). Pumps were filled either with recombinant murine VEGF (Peprotech, Hamburg, Germany) diluted in phosphate-buffered saline (PBS) with 0.1% bovine serum albumin (BSA) or with PBS with 0.1% BSA as a control. The delivery rate was set at 75 ng/h. Five-week-old mice were fed an HFD for 5 weeks before pump implantation; after pump implantation, mice were fed for additional 3 weeks prior to euthanasia and further analysis.

### Protein detection.

Adipose tissues (AT) were excised after euthanasia and proteins isolated as described elsewhere (34). Briefly, AT were homogenized and digested in radioimmunoprecipitation assay (RIPA) lysis buffer (1% Triton X-100; 0.5% sodium deoxycholate; 0.1% SDS; 50 mM Tris-HCl, pH 7.5; 150 mM NaCl; mini-protease inhibitor and phosphatase inhibitor cocktail tablet [Roche]), incubated on ice for 20 min, and centrifuged to remove cellular debris and fat. Protein concentration was determined using a bicinchoninic acid (BCA) protein assay kit (Thermo Scientific, Schwerte, Germany). Antibodies (Abs) against UCP1 (Abcam, Cambridge, United Kingdom), phospho-Ser473 Akt, total Akt (Cell Signaling/New England BioLabs, Frankfurt am Main, Germany), and tubulin (Sigma-Aldrich) were used for immunoblotting. For blot quantification, densitometry was performed with ImageJ software (National Institutes of Health, Bethesda, MD); tubulin was used for UCP1 normalization, whereas total Akt was used for phospho-Ser473 Akt normalization.

VEGF-A was determined in WAT and BAT lysates using an ELISA kit (R&D Systems).

### Gene expression.

Total RNA from tissues or cells was isolated using TRIzol (Invitrogen, Darmstadt, Germany) by following the manufacturer's instructions. After purification with DNase I treatment (Thermo Scientific), 1 μg of RNA was reverse transcribed using an iScript cDNA synthesis kit (Bio-Rad, Munich, Germany), and real-time PCR was performed with SsoFast EvaGreen Supermix (Bio-Rad) using a Bio-Rad CFX384 Touch real-time PCR detection system (Bio-Rad). Calculation was based on the threshold cycle (ΔΔ*C_T_*) method (35), and normalization to 18S RNA was performed.

### *Ex vivo* lipolysis assay in adipose tissue explants.

Subcutaneous and gonadal fat depots (scWAT and gonWAT, respectively) were surgically removed from 24-week HFD-fed control and AdHIF2KO mice and washed with ice-cold PBS. A piece of 100 mg was excised, cut into 5 or 6 pieces, and incubated for 2 h at 37°C in 250 μl of Dulbecco modified Eagle medium (DMEM) containing 2% fatty acid-free BSA (Sigma-Aldrich) and in the presence or absence of 10 μM isoprenaline (Sigma-Aldrich). Fatty acids released to the medium were quantified using a free fatty acid fluorometric assay kit (Cayman Chemical).

### Cell culture.

For the isolation of bone marrow-derived macrophages (BMDM), we followed our previously published protocol (36).

### Histology.

Fresh tissues were excised, fixed in 4% paraformaldehyde, and paraffin embedded. Sections were stained with hematoxylin and eosin (H&E). Histological scoring for liver nonalcoholic fatty liver disease (NAFLD)/nonalcoholic steatohepatitis (NASH) was read blinded to the experimental design using H&E staining. The degree of steatosis, ballooning, and lobular inflammation was evaluated according to previously published criteria by following the NASH-CRN Committee scoring system (37, 38). The NAFLD activity score (NAS) consists of the sum of steatosis, ballooning, and lobular inflammation. A NAS of >5 correlates with the presence of NASH (38). For immunohistochemistry, sections were deparaffinized and antigen retrieval was done by incubation with hot citrate buffer. Inhibition of intrinsic peroxidase activity was performed with 0.5% H_2_O_2_ treatment. In addition, F4/80 staining required proteinase K (Sigma-Aldrich) treatment at 37°C, whereas for UCP1 staining, sections were also incubated with pronase (Sigma-Aldrich) at 37°C. Sections were permeabilized with 0.4% Triton X-100 and blocked with serum from an ABC kit (Vector Laboratories, Peterborough, United Kingdom). Sections were then incubated overnight with antibodies against UCP1 (Abcam), or against F4/80 (Novus Biologicals, Herford, Germany). The avidin-biotin complex was detected with a 3-amino-9-ethylcarbazole (AEC) peroxidase substrate kit (Vector Laboratories). F4/80 quantification was performed by counting the F4/80-positive cells per adipocyte per field in at least 6 random low-magnification fields per sample. Terminal deoxynucleotidyltransferase-mediated dUTP-biotin nick end labeling (TUNEL) staining was performed using a Dead End colorimetric TUNEL kit by following the manufacturer's instructions (Promega, Mannheim, Germany). TUNEL quantification was performed by counting the TUNEL-positive cells per adipocyte per field in at least 6 random low-magnification fields per sample. Pictures were visualized by a computerized microscope (Zeiss, Oberkochen, Germany) and analyzed with AxioVision Rel 4.8 software (Zeiss). For oil red O staining, 10-μm cryosections were prepared. Slides were fixed in 4% paraformaldehyde for 1 h, rinsed in distilled water (dH_2_O), and stained for 15 min in oil red O in 60% isopropanol. They were rinsed with 60% isopropanol and counterstained with Mayer's hematoxylin. Quantification was performed as described previously (39). Fibrosis staining was performed by using a Masson trichrome staining kit (Sigma-Aldrich) according to the manufacturer's instructions. For hypoxia staining, mice were intraperitoneally injected with 60 mg/kg of CCl-103F (Hypoxyprobe F6; Hypoxyprobe, Burlington, MA) and sacrificed 90 min after the injection. Tissues were fixed and processed as described above using a Hypoxyprobe F6 kit (Hypoxyprobe) by following the manufacturer′s instructions; quantification was performed by determining the percentage of the area staining positive for hypoxia in 5 random low-magnification fields per sample. AT whole-mount staining was performed by a previously described method (40), with some modifications. Briefly, WAT and BAT were fixed with paraformaldehyde and cut in small pieces (2 mm by 2 mm). After blocking and permeabilization with BSA (1%) and Triton X-100 (0.5%), mounts were stained with fluorescein isothiocyanate (FITC)-conjugated isolectin B4 (Sigma-Aldrich) at 4°C. After several washings with PBS-Tween (PBST), samples were visualized using a confocal microscope (Leica TCS SP5; Leica, Wetzlar, Germany). Z-stacks of a 5-μm depth were analyzed and quantification of fluorescence intensity was performed using LAS software (Leica).

### Measurement of liver triglyceride content.

Triglyceride content quantification in the liver was done using a commercially available kit (Abcam). Briefly, 100 mg of liver tissue was homogenized in 1 ml of 5% Triton X-100. The samples were then heated to 95°C and cooled to room temperature twice. Thereafter, samples were centrifuged, and triglyceride content in the supernatant was quantified using enzymatic determination.

### Flow cytometry.

After mice were sacrificed, scWAT and gonWAT were excised and the lymph nodes, immersed in the fat depot, were removed. AT was then digested using collagenase type I (2 mg/ml per g of tissue; Life Technologies) for 60 min at 37°C. The suspension was resuspended in DMEM containing 0.5% fatty acid-poor BSA (Sigma-Aldrich) and centrifuged to separate the floating adipocyte fraction from the pelleted stromal vascular fraction (SVF). For fluorescence-activated cell sorter (FACS) analysis, the following antibodies were used: Fc receptor-blocking Ab 2.4G2, CD45-Alexa Fluor 488 (Biolegend, Fell, Germany), and CD31-allophycocyanin (CD31-APC; eBioscience, Frankfurt, Germany). FACS was carried out on a FACSCanto II (BD, Heidelberg, Germany) and analyzed with FACSDiva version 6.1.3 software.

Sorting of lung endothelial cells from endothelium-specific HIF2α KO mice was performed as previously described (32). The solution was filtered and stained for CD31 (eBioscience), and sorting for CD31-positive and CD31-negative cells was performed using a FACS Aria II sorter (BD).

### Metabolic cage analysis.

Metabolic cage analysis was done with mice that were fed an HFD for 6 weeks. Mice were individually housed in metabolic cages (PhenoMaster; TSE Systems, Bad Homburg, Germany) with free access to water and food, maintaining a 12 h:12 h light-dark cycle. A period of at least 16 h of acclimatization in the metabolic cages preceded initiation of the experiment and data collection. Volume of oxygen consumption (VO_2_) and carbon dioxide production (VCO_2_) were determined every 20 min. The respiratory exchange ratio (RER) was calculated as VCO_2_/VO_2_. Energy expenditure (EE) was calculated as (3.941 × VO_2_) + (1.106 × VCO_2_) (41). Food intake was also monitored. Data were normalized with respect to body weight using analysis of covariance (ANCOVA).

### Statistical analyses.

Data are expressed as means ± standard errors of the means (SEMs) and were statistically analyzed by Student's *t* test or Mann-Whitney U test as appropriate. Body temperature during cold exposure experiment was analyzed by analysis of variance (ANOVA). Significance was set at a *P* value of <0.05.

## RESULTS

### Adipocyte HIF2α deficiency promotes HFD-induced metabolic dysregulation.

**FIG 1:**
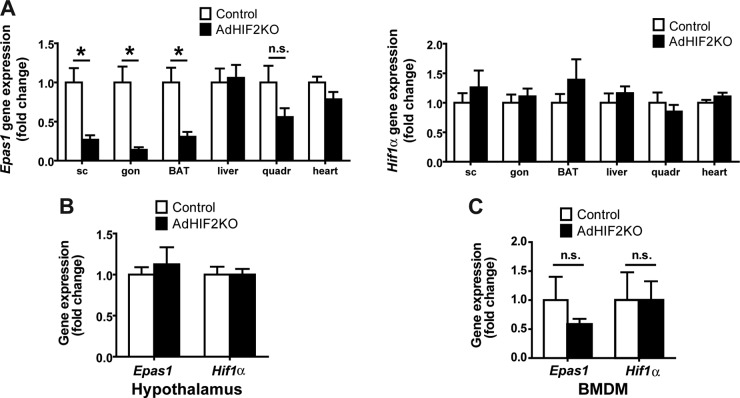
HIF2α deletion in AdHIF2KO mice. (A) *Epas1* (*Hif2*α) (left) and *Hif1*α (right) gene expression in subcutaneous (sc) and gonadal (gon) white AT (WAT), brown AT (BAT), liver, quadriceps skeletal muscle, and heart (*n =* 5 to 8/group). Gene expression of control mice was set as 1. (B and C) *Epas1* and *Hif1*α gene expression in hypothalamus (B) and bone marrow-derived macrophages (BMDM) (C). Gene expression of control mice was set as 1 (*n =* 3 or 4/group). Data are expressed as means ± SEMs. *, *P* < 0.05.

Mice with adipocyte-specific deletion of HIF2α were generated by crossing mice with a floxed *Epas1* (HIF2α) allele with mice expressing Cre recombinase under the control of the Fabp4 promoter. This resulted in Fabp4Cre^+^ *Epas1*^fl/fl^ mice, in which HIF2α was deleted in white and brown adipocytes (AdHIF2KO) (Fig. 1A) (42), while Fabp4Cre^−^ *Epas1*^fl/fl^ mice were used as littermate HIF2α-proficient mice (referred to as control mice). Efficient deletion of HIF2α was observed in subcutaneous WAT (scWAT) and visceral gonadal WAT (gonWAT) and in BAT, whereas HIF2α expression was unaffected in nonadipose tissues and cells such as the liver, skeletal muscle, heart, hypothalamus, and bone marrow-derived macrophages (BMDM) (Fig. 1). HIF1α expression was not affected by adipocyte-specific HIF2α deletion in any of these tissues (Fig. 1).

**FIG 2:**
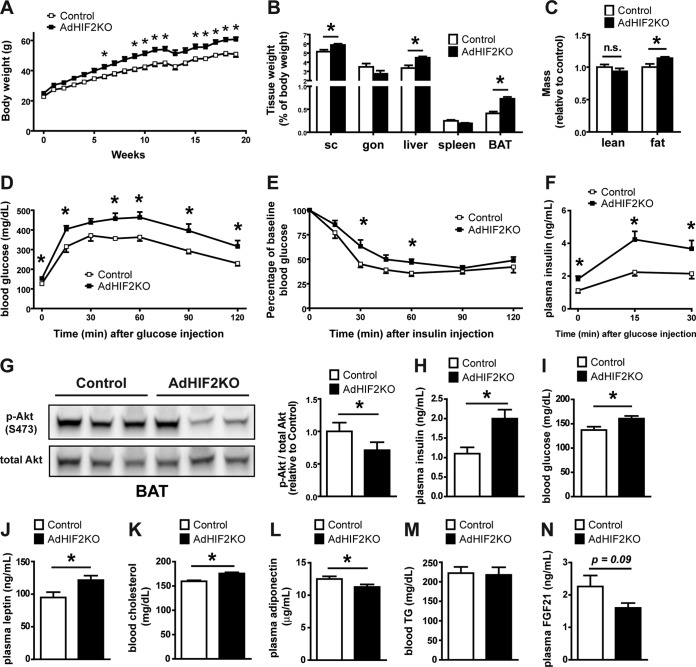
Mice lacking HIF2α in adipocytes display worsened obesity-related metabolic dysregulation. (A) Body weight of control and AdHIF2KO mice subjected to an HFD. (B) Mice were sacrificed after 24 weeks on an HFD, and subcutaneous (sc) and gonadal (gon) WAT, liver, spleen, and BAT were weighed. The tissue weights are presented as percentages of total body weight. (C) Lean and fat body mass was determined in obese (24 weeks on an HFD) control and AdHIF2KO mice by computed tomography (CT); data were normalized to those for control mice. (D) Glucose tolerance test of control and AdHIF2KO mice fed with an HFD for 12 weeks. (E) Insulin tolerance test of control and AdHIF2KO mice fed with an HFD for 14 weeks. (F) Glucose-stimulated insulin secretion of control and AdHIF2KO mice fed with an HFD. (G) Control and AdHIF2KO mice were fed with an HFD for 15 weeks. Mice were fasted for 6 h before an intraperitoneal injection of insulin (5 U/kg of body weight), and BAT was harvested and analyzed for phosphorylation of Akt at Ser473 and total Akt. Representative blots (left) and densitometric quantification of the blots (right panel) are shown. Data (densitometric analysis) are expressed as means ± SEMs (*n =* 6 mice per group). (H to N) Plasma insulin (H), blood glucose (I), plasma leptin (J), blood cholesterol (K), plasma adiponectin (L), blood triglycerides (M), and plasma FGF-21 (N) of mice of the indicated genotypes fed for 15 weeks with an HFD and fasted overnight. In panels A to F and H to N, data are expressed as means ± SEMs (*n =* 6 to 15 mice/group). *, *P* < 0.05.

AdHIF2KO and control mice were fed an HFD or ND. Mice lacking HIF2α in adipocytes showed a significant increase in HFD-induced body weight gain (Fig. 2A), accompanied by increased weight of the scWAT, liver, and BAT after 16 and 24 weeks on the HFD (Fig. 2B, data from mice fed an HFD for 24 weeks; data from mice fed an HFD for 16 weeks are not shown). In contrast, the weight of gonWAT did not differ between HFD-fed AdHIF2KO mice and HFD-fed control mice. The increase in the mass of scWAT, liver, and BAT was not simply due to enhanced body weight, since a significant increase in the weight of these tissues was also found when weights were plotted as percentage of total body weight (Fig. 2B, data from mice fed an HFD for 24 weeks; data from mice fed an HFD for 16 weeks are not shown). In keeping with these data, fat body mass determined by computed tomography (CT) was elevated in AdHIF2KO mice compared to that in control mice, whereas no difference was found in lean body mass between both genotypes (Fig. 2C).

**FIG 3:**
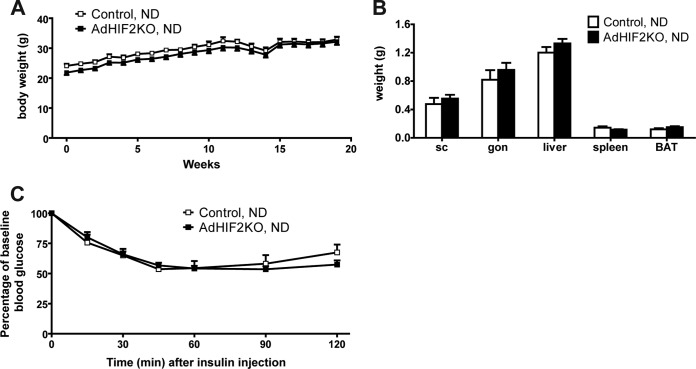
Lean AdHIF2KO mice do not show metabolic dysregulation. (A) Body weights of control and AdHIF2KO mice fed with a normal diet (ND) (10% kilocalories from fat). (B) Weights of subcutaneous (sc) and gonadal (gon) WAT, liver, spleen, and BAT from control and AdHIF2KO mice fed with an ND for 24 weeks. (C) Insulin tolerance test of control and AdHIF2KO mice fed with an ND for 13 weeks. Data are expressed as means ± SEMs (*n =* 6 to 9 mice/group).

AdHIF2KO mice on an HFD showed increased glucose intolerance and insulin resistance compared to those of control mice (Fig. 2D and E). Analysis of glucose-stimulated insulin secretion (GSIS) demonstrated elevated insulin secretion upon glucose administration in AdHIF2KO mice (Fig. 2F), reflecting the impaired insulin sensitivity. Furthermore, insulin signaling was assessed, and BAT of AdHIF2KO mice exhibited reduced Akt phosphorylation in response to insulin administration compared to that in control mice (Fig. 2G). In contrast, insulin-induced Akt phosphorylation was not different in scWAT and gonWAT, liver, and muscle due to adipocyte HIF2α deficiency (data not shown). Fasting plasma insulin, glucose, leptin, and cholesterol levels were enhanced in obese AdHIF2KO mice, while adiponectin levels were significantly lower in obese AdHIF2KO mice than in control mice (Fig. 2H to L). In contrast, there was no significant difference in blood triglycerides or FGF-21 (43) in AdHIF2KO mice (Fig. 2M and N). When fed an ND, AdHIF2KO mice displayed no significant changes in body weight, fat mass, or insulin sensitivity compared to those in control mice (Fig. 3). These findings indicate that adipocyte HIF2α contributes to the adaptive response of WAT to obesity and protects against HFD-induced metabolic dysregulation.

**FIG 4:**
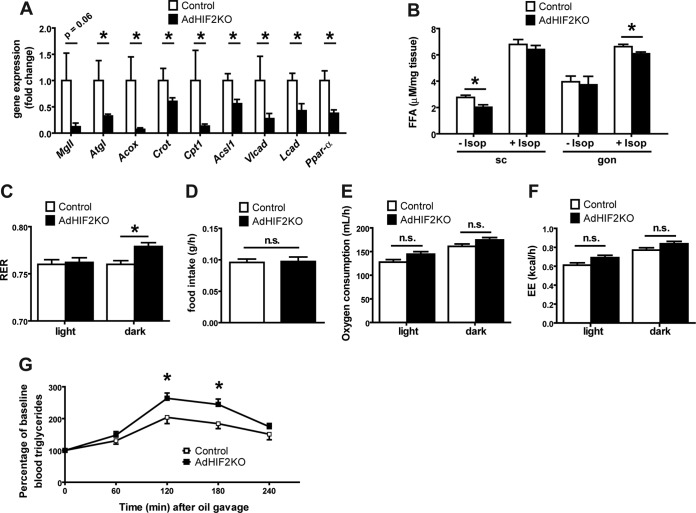
Obese AdHIF2KO mice show altered lipid metabolism. (A) Expression of metabolic genes in gonadal adipocyte fraction from control and AdHIF2KO mice fed an HFD for 24 weeks is shown. Gene expression of control mice was set as 1 (*n =* 6 or 7/group). (B) Subcutaneous (sc) WAT and gonadal (gon) WAT explants from obese control and AdHIF2KO mice were cultured for 2 h at 37°C in the presence or absence of 10 μM isoprenaline, and release of FFA to the medium was quantified (*n =* 4 to 6/group). (C to F) Control and AdHIF2KO mice were fed with an HFD for 6 weeks and placed in metabolic cages. After 16 h of adaptation, respiratory exchange ratio (RER) (C), food intake (D), oxygen consumption (E), and energy expenditure (EE) (F) were monitored (*n =* 6/group). (G) Oral lipid tolerance test of control and AdHIF2KO mice fed with an HFD for 15 weeks (*n =* 12 or 13/group). Data are expressed as means ± SEMs. *, *P* < 0.05.

We then analyzed the alterations in WAT induced by adipocyte HIF2α deficiency. We found that the mRNA expression of the adipocyte lipase *Atgl* was significantly reduced in the gonWAT of obese AdHIF2KO mice; moreover, the expression of the lipase *MgII* was lower (although not significantly) than in obese control mice (Fig. 4A). In the gonWAT of AdHIF2KO mice, we also found decreased expression of genes involved in peroxisomal (*Acox* and *Crot*) and mitochondrial (*Cpt1* and *Acsl1*) lipid oxidation and of genes involved in β-oxidation of long-chain fatty acids (*Vlcad* and *Lcad*) and reduced expression of the lipolysis regulator (*Ppar*-α) owing to adipocyte HIF2α deficiency (Fig. 4A). In addition, scWAT showed reduced expression of genes involved in lipolysis (*MgII*) and peroxisomal lipid oxidation (*Acox*) (data not shown). Explants of scWAT from obese AdHIF2KO mice showed reduced basal lipolysis, whereas gonWAT explants from obese AdHIF2KO mice displayed diminished isoprenaline-stimulated lipolysis (Fig. 4B) compared to that in respective explants from obese control mice. Furthermore, we found elevated RER in obese AdHIF2KO mice, indicating reduced lipid consumption, compared to that in obese control mice, while the food intake, oxygen consumption, and energy expenditure (EE) were not affected by adipocyte HIF2α deficiency (Fig. 4C to F). In addition, AdHIF2KO mice showed elevated triglyceride levels in an oral lipid tolerance test (Fig. 4G), suggesting defective lipid metabolism due to adipocyte HIF2α deficiency.

### Adipocyte HIF2α deficiency decreases WAT angiogenesis and enhances WAT inflammation in obesity.

**FIG 5:**
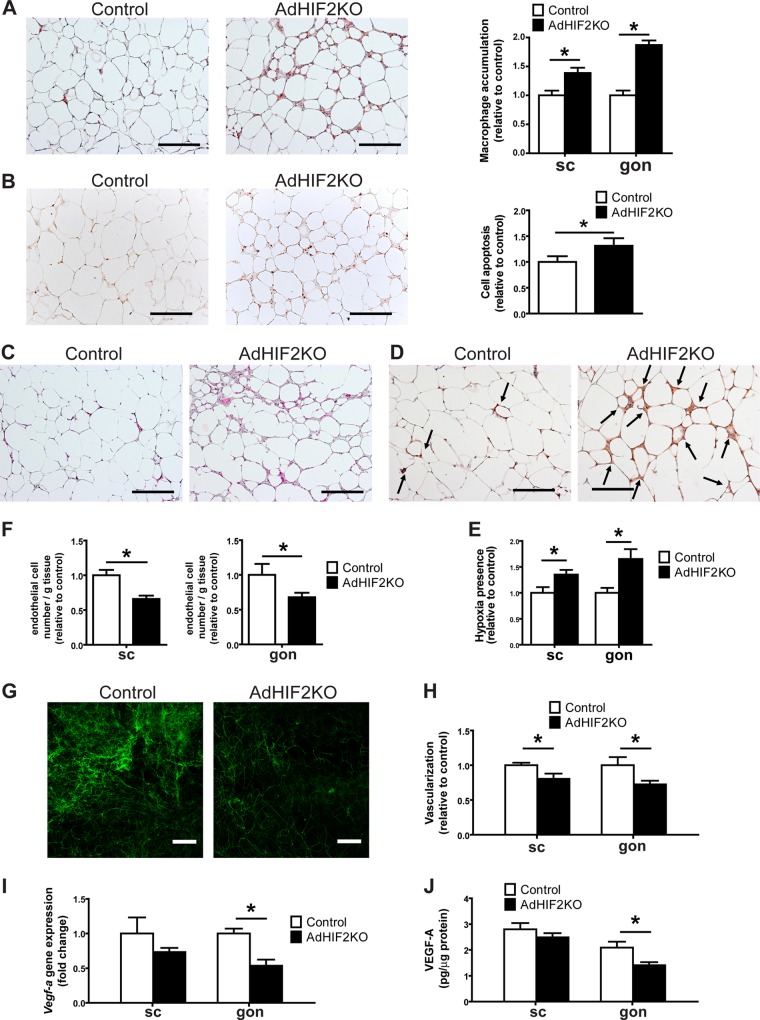
Obese AdHIF2KO mice show enhanced WAT inflammation and reduced WAT vascularity. (A to C) Mice were sacrificed after 24 weeks on an HFD. (A) Representative pictures in gonadal (gon) WAT (left) and quantification from subcutaneous (sc) and gon (right) WAT of immunohistochemistry for F4/80 (*n =* 5/group). Macrophage accumulation of control mice was set as 1. Scale bars are 200 μm. (B) Representative pictures of TUNEL immunohistochemistry from the gonWAT of obese control and AdHIF2KO mice (24 weeks on an HFD) and the respective quantification (right) are shown (*n =* 5/group). WAT apoptosis of control mice was set as 1. Scale bar is 200 μm. (C) Representative pictures of Masson trichrome staining for fibrosis detection in gonWAT from control and AdHIF2KO mice. Scale bars are 200 μm. (D and E) Representative images of staining for CCl-103F (Hypoxiprobe-F6) in gonWAT (D) as well as quantification of hypoxia staining (E) from scWAT and gonWAT from obese control and AdHIF2KO mice (16 weeks on an HFD) are shown (*n =* 5 or 6/group). Arrows indicate hypoxic areas. Scale bars are 200 μm. (F to J) Mice were sacrificed after 24 weeks on an HFD. (F) scWAT and gonWAT were digested with collagenase, and flow cytometry analysis for CD31^+^ CD45^−^ cells was performed to analyze endothelial cell numbers (*n =* 10 to 17). The absolute endothelial cell number per gram of tissue was quantified. Data are shown relative to control; data of control mice were set as 1. (G and H) Representative images of scWAT (G) of isolectin B4 staining in whole mounts and quantification (H) of isolectin B4 staining in whole mounts from scWAT and gonWAT (*n =* 5 to 7). Vascularization of control mice was set as 1. Scale bars are 200 μm. (I) *Vegf-a* gene expression in subcutaneous and gonadal adipocyte fractions from control and AdHIF2KO mice (*n =* 6 or 7). Gene expression of control mice was set as 1. (J) VEGF-A protein levels measured in scWAT and gonWAT lysates from control and AdHIF2KO mice, normalized over total protein content (*n =* 4 to 7). Data are expressed as means ± SEMs. *, *P* < 0.05.

Given the strong link between insulin resistance and AT inflammation (44–46), we sought to determine the inflammatory status of the obese WAT in HIF2α deficiency. Immunohistochemical staining for F4/80, a marker for macrophages, showed increased abundance of macrophages in the gonWAT and scWAT of obese AdHIF2KO mice compared to that in control mice (Fig. 5A). AT inflammation is associated with formation of crown-like structures, found mostly in gonWAT, consisting of apoptotic adipocytes surrounded by macrophages (47). Besides higher macrophage accumulation, we found more apoptotic cells, as measured by TUNEL immunohistochemistry in the gonWAT of obese AdHIF2KO mice (Fig. 5B). Furthermore, WAT dysfunction is linked to WAT fibrosis, which occurs by macrophage-mediated remodeling of the extracellular matrix (34, 48). By Masson′s trichrome staining, which is specific for collagen deposition, we found increased fibrosis of the gonWAT in obese AdHIF2KO mice (Fig. 5C). Together, our findings show that adipocyte HIF2α protects against obesity-mediated WAT inflammation and fibrosis and, thus, WAT dysfunction.

Adipocyte hypertrophy in obesity leads to hypoxic areas in the WAT of men and mice (4, 5). AT hypoxia stimulates vascular growth in the AT (8). Interestingly, WAT of obese AdHIF2KO mice displayed marked hypoxia compared to the WAT of obese control mice (Fig. 5D and E). Limited vascularization of WAT is linked to elevated presence of hypoxia, as well as insulin resistance, enhanced AT inflammation, and adipocyte death (8). On the other hand, enhanced vascularization of AT prevents obesity-related metabolic dysregulation of AT (8, 11). HIF2α is a major regulator of proangiogenic responses in several physiological and pathological situations (18, 49, 50). We found reduced WAT vascularization in obese AdHIF2KO mice compared to that in control mice, which was reflected by reduced numbers of endothelial cells in both WAT depots, as assessed by flow cytometry (Fig. 5F). Moreover, vascularization was studied by staining with isolectin B4 and was found to be decreased in the scWAT and gonWAT of obese AdHIF2KO mice compared to that in controls after 16 and 24 weeks on an HFD (Fig. 5G and H, data from mice fed an HFD for 24 weeks; data from mice fed an HFD for 16 weeks are not shown). A major HIF2α target regulating angiogenesis is VEGF-A (18, 49, 50). Consistently, its expression was reduced in the gonWAT of obese AdHIF2KO mice (Fig. 5I and J). Thus, the absence of HIF2α resulted in insufficient angiogenic response in the obese WAT, thereby contributing to enhanced WAT hypoxia, inflammation, and cell death.

### No role of HIF2α in myeloid or endothelial cells for WAT adaptation to obesity.

**FIG 6:**
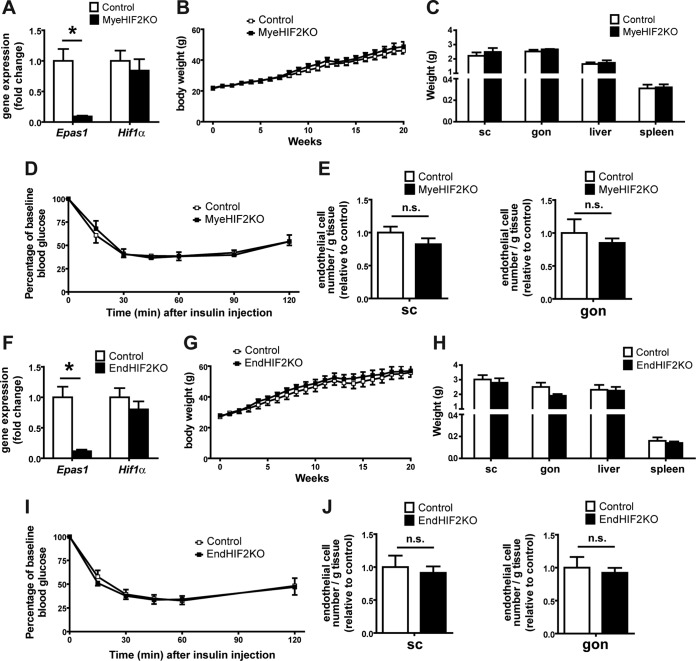
No role of myeloid or endothelial HIF2α in obesity-related metabolic dysregulation. (A) Effective deletion of *Epas1* (*Hif2*α) but not of *Hif1*α in bone marrow-derived macrophages from control and MyeHIF2KO mice. Gene expression of control mice was set as 1. (B) Body weights of HFD-fed control and MyeHIF2KO mice. (C) Tissue weights of obese control and MyeHIF2KO mice. (D) Insulin tolerance test of obese MyeHIF2KO and control mice. (E) FACS staining for endothelial cells (CD31^+^ CD45^−^) in the SVF of subcutaneous (sc) and gonadal (gon) WAT of obese MyeHIF2KO and littermate control mice. The absolute endothelial cell number per gram of tissue was quantified. Data are shown relative to those for the control; data of control mice were set as 1. For panels A to E, data are expressed as means ± SEMs (*n =* 4 or 5 per group). *, *P* < 0.05. n.s., not significant. (F) Effective deletion of *Epas1* (*Hif2*α) but not of *Hif1*α was assessed in sorted lung endothelial cells from control and EndHIF2KO mice. Gene expression of control mice was set as 1. (G) Body weights of HFD-fed control and EndHIF2KO mice. (H) Tissue weights of obese EndHIF2KO and littermate control mice. (I) Insulin tolerance test of obese EndHIF2KO and littermate control mice. (J) FACS staining for endothelial cells (CD31^+^ CD45^−^) from the SVF of scWAT and gonWAT of obese EndHIF2KO and littermate control mice. The absolute endothelial cell number per gram of tissue was quantified. Data are shown relative to those for the control; data of control mice were set as 1. For panels F to J, data are expressed as means ± SEMs (*n =* 5 per group). *, *P* < 0.05.

Our findings so far indicate that adipocyte HIF2α deletion contributes to obese WAT dysfunction, including reduced vascularization and enhanced inflammation in obese WAT, thereby resulting in insulin resistance. A major expression of HIF2α is found in myeloid and endothelial cells, and HIF2α in these cells is capable of regulating angiogenic responses (50, 51). As Fabp4-Cre transgenic mice have been previously reported to potentially delete target genes in nonadipocytes, e.g., in macrophages (13, 52, 53), we sought to clarify if myeloid HIF2α played any role in the WAT phenotype observed in obese AdHIF2KO mice. To this end, we generated myeloid cell-specific HIF2α-deficient mice (MyeHIF2KO) by engaging LysM-Cre transgenic mice. Compared to their littermate controls, mice lacking HIF2α in myeloid cells did not show any metabolic changes in diet-induced obesity, as assessed by analysis of body weight and fat mass and the insulin tolerance test (Fig. 6A to D). These data suggest that myeloid HIF2α does not substantially contribute to obesity-related WAT dysfunction.

Moreover, HIF2α in endothelial cells regulates angiogenesis-related functions (51). To assess the role of endothelial HIF2α, we generated endothelial-cell-specific HIF2KO mice (EndHIF2KO) and subjected them to diet-induced obesity. In contrast to AdHIF2KO mice, mice lacking HIF2α in endothelial cells did not develop any metabolic alterations compared to littermate HIF2α-proficient mice (Fig. 6F to I). Importantly, the number of endothelial cells in the obese WAT did not change in either MyeHIF2KO or EndHIF2KO mice (Fig. 6E and J) compared to that in control littermate mice, thus indicating that obese WAT angiogenesis is regulated predominantly by adipocyte HIF2α.

### Ectopic accumulation of fat in adipocyte HIF2α-deficient mice.

**FIG 7:**
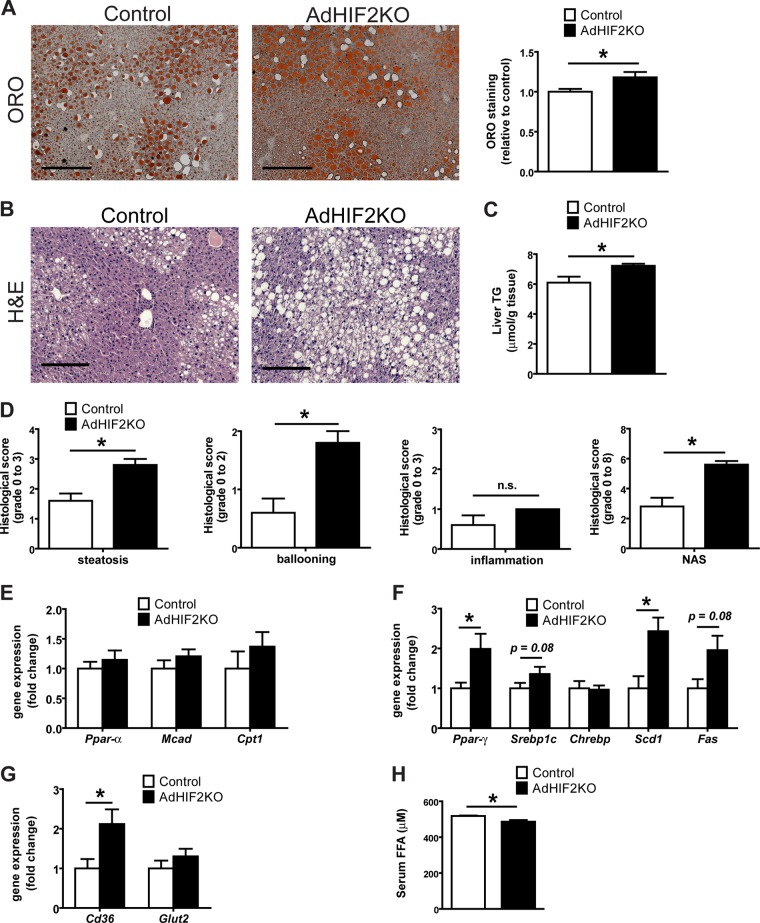
Obese AdHIF2KO mice develop enhanced hepatosteatosis. Mice were sacrificed after 24 weeks on an HFD. (A) Representative pictures of oil red O (ORO) staining of livers from control and AdHIF2KO mice and quantification (*n =* 5/group). Steatosis (ORO staining) of control mice was set as 1. Scale bar is 200 μm. (B) Representative images (H&E staining) of obese control and AdHIF2KO mouse livers. Scale bars are 200 μm. (C) Triglyceride content was determined in livers from control and AdHIF2KO mice fed for 24 weeks with an HFD (*n =* 6 or 7). (D) Histological scoring for steatosis, hepatocellular ballooning, lobular inflammation and NAFLD activity score (NAS) of livers from obese control and AdHIF2KO mice (*n =* 5). (E to G) Gene expression analysis for lipolytic (E) and lipogenic markers (F), as well as transporters for FFA (*Cd36*) and glucose (*Glut2*) (G) in livers from obese control and AdHIF2KO mice (*n =* 6 or 7). Gene expression of control mice was set as 1. (H) Serum free fatty acids (FFA) from control and AdHIF2KO mice fed an HFD (*n =* 8 to 12). Data are expressed as means ± SEMs. *, *P* < 0.05.

Deficient lipolytic activity of WAT has been linked to ectopic accumulation of fat (e.g., in the liver) along with reduced insulin sensitivity (10, 54). Livers of obese AdHIF2KO mice showed not only increased size (Fig. 2B) but also increased lipid accumulation. As assessed by histology analysis, we found enhanced abundance of lipid droplets in livers due to adipocyte HIF2α deficiency in mice fed an HFD for 16 or 24 weeks (Fig. 7A and B, data from mice fed an HFD for 24 weeks; data from mice fed an HFD for 16 weeks are not shown). This finding was confirmed by measuring liver triglyceride content, which was higher in AdHIF2KO mice (Fig. 7C). By histological assessment, AdHIF2KO mice displayed enhanced steatosis and ballooning and an enhanced NAFLD activity score (NAS) (Fig. 7D); a NAS of >5, as observed in AdHIF2KO mice, correlates with the presence of NASH (38). Gene expression analysis showed unaltered expression of lipolytic markers, such as *Ppar*-α, *Mcad*, and *Cpt1* (Fig. 7E), but significantly increased expression of lipogenic markers, such as *Ppar*-γ and *Scd1*, as well as a tendency toward increased expression of *Srebp1c* and *Fas* in livers from obese AdHIF2KO mice compared to that in littermate control mice (Fig. 7F). These findings could explain the enhanced steatosis observed in adipocyte HIF2α deficiency under obese conditions. While the expression of the main hepatic glucose transporter gene, *Glut2*, was unchanged, fatty acid transporter gene (*Cd36*) expression was significantly increased in livers of obese AdHIF2KO mice, thereby suggesting enhanced uptake of lipids by the liver (Fig. 7G). The enhanced hepatic lipid uptake was also in keeping with reduced serum free fatty acid (FFA) levels in obese AdHIF2KO mice (Fig. 7H).

### Deficiency of adipocyte HIF2α leads to BAT dysfunction in obesity.

We next analyzed the BAT of AdHIF2KO mice, as BAT is one of the main targets of the activity of Cre recombinase in Fabp4-Cre transgenic mice (Fig. 1A). The main function of BAT is heat generation via lipid consumption in brown adipocytes. Recently, BAT has gained attention in the context of obesity (reviewed in reference 23); with the progression of obesity, BAT gains weight and accumulates lipids and brown adipocytes become bigger, switching from the typical brown appearance (several lipid droplets per cell) to a “white fat-like” shape comprising a huge fat drop.

**FIG 8:**
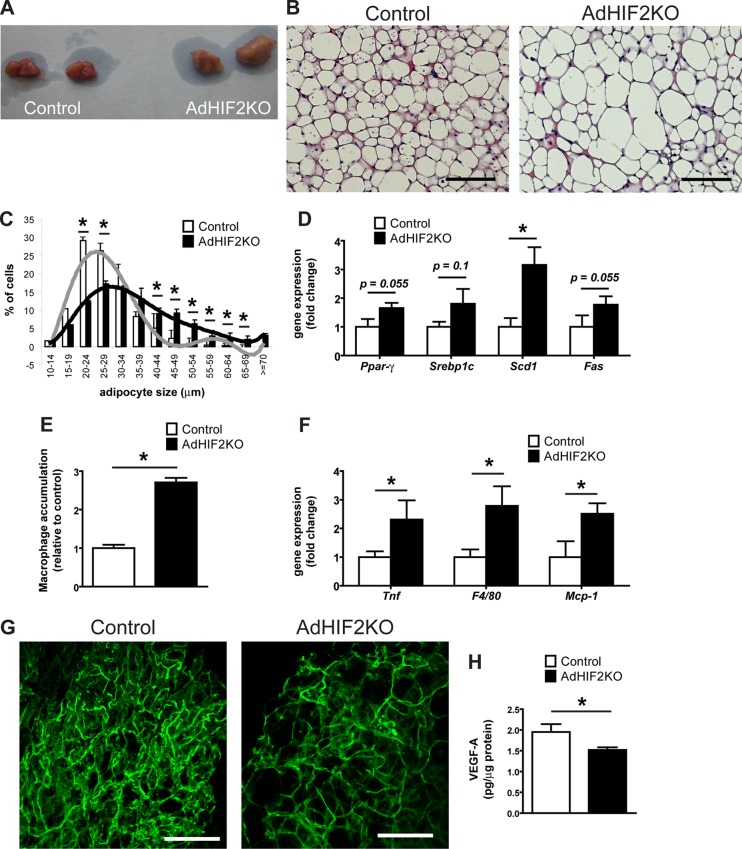
Dysfunction of BAT in obese AdHIF2KO mice. (A) Representative images of BAT of obese control and AdHIF2KO mice. (B) Representative H&E staining of BAT from control and AdHIF2KO mice upon HFD for 24 weeks. Scale bars are 100 μm. (C) Quantification of brown adipocyte cell diameter and fitting curve of control (gray line) and AdHIF2KO (black line) mice. (D) Gene expression analysis for lipogenic markers in BAT from obese control and AdHIF2KO mice fed an HFD for 24 weeks. Gene expression of control mice was set as 1. (E) Quantification of F4/80 immunohistochemistry in BAT from control and AdHIF2KO mice after being fed an HFD for 24 weeks. Macrophage accumulation of control mice was set as 1. (F) Gene expression of proinflammatory markers in BAT from control and AdHIF2KO mice on an HFD for 24 weeks. Gene expression of control mice was set as 1. (G) Representative images of isolectin B4 staining in whole mounts from BAT of control and AdHIF2KO mice on an HFD for 24 weeks. Scale bars are 100 μm. (H) VEGF-A protein levels, normalized over total protein content, were measured in BAT lysates from control and AdHIF2KO mice on an HFD for 24 weeks. Data in panels C to F and H are expressed as means ± SEMs (*n =* 5 to 7 mice per group). *, *P* < 0.05.

As described above (Fig. 2B), the BAT of mice lacking HIF2α in adipocytes displayed higher mass. In addition to its enhanced mass, the BAT of obese AdHIF2KO mice displayed a marked pale color, suggesting lipid accumulation (Fig. 8A). Microscopic analysis of the BAT revealed that brown adipocytes from HFD-fed AdHIF2KO mice were larger than those in littermate control mice (Fig. 8B and C, data from mice fed an HFD for 24 weeks; data from mice fed an HFD for 16 weeks are not shown). Interestingly, this was not observed under ND conditions (data not shown). Accordingly, BAT from HFD-fed AdHIF2KO mice showed enhanced expression of lipogenic markers (Fig. 8D), suggesting that excessive lipogenesis could contribute to the elevated cell size of brown adipocytes in HIF2α deficiency.

Consistent with recent reports with regard to induction of inflammation in the BAT during obesity (55, 56), we found that dysfunction of the BAT in obese AdHIF2KO mice was linked to higher BAT inflammation, as assessed by increased accumulation of F4/80-positive macrophages (Fig. 8E, data from mice fed an HFD for 24 weeks; data from mice fed an HFD for 16 weeks are not shown). Moreover, expression of *F4/80* and other inflammatory factor genes, such as *Tnf* or *Mcp-1*, was upregulated in the BAT of obese AdHI2KO mice compared to that in littermate control mice (Fig. 8F).

BAT function largely depends on angiogenesis (3, 57); by staining with fluorochrome-labeled isolectin B4, we found reduced vascularity in the BAT of obese AdHIF2KO mice (Fig. 8G) compared to that in littermate control mice, suggesting that HIF2α supports angiogenic processes in BAT in obesity. Consistently, VEGF-A levels were downregulated in the BAT of obese AdHIF2KO mice (Fig. 8H). Together, these data demonstrate that HIF2α is necessary for promoting angiogenesis not only in WAT but also in BAT in obesity, by regulating BAT VEGF-A levels.

**FIG 9:**
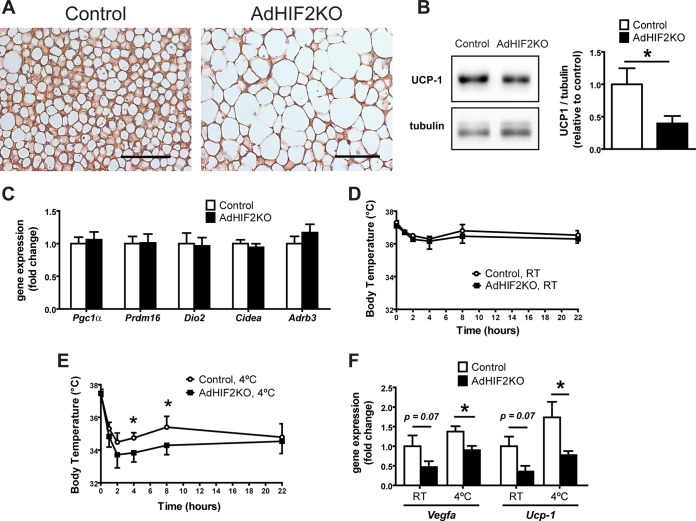
Reduced UCP1 expression in the BAT of obese adipocyte-specific HIF2α-deficient mice. (A) Representative images of UCP1 immunohistochemistry in BAT from control and AdHIF2KO mice fed for 24 weeks on an HFD diet. Scale bars are 100 μm. (B) Representative Western blot for UCP1 and tubulin (left) and quantification (right) from BAT protein lysates from control and AdHIF2KO mice after being fed an HFD for 24 weeks. The UCP1/tubulin ratio of control mice was set as 1 (*n =* 4 or 5/group). (C) Gene expression of *Pgc1*α, *Prdm16*, *Dio2*, *Cidea*, and *Adrb3* in BAT from control and AdHIF2KO mice fed an HFD for 24 weeks. Gene expression of control mice was set as 1 (*n =* 7 to 14/group). (D and E) Obese control and AdHIF2KO mice were exposed to either room temperature (RT) (D) or 4°C (E), and temperature was measured (*n =* 3 or 4 for RT and *n =* 9 to 12 for 4°C). (F) Gene expression analysis of the BAT from the mice whose results are displayed in panels D and E. Gene expression of control RT mice was set as 1. Data in panels B to F are expressed as means ± SEMs. *, *P* < 0.05.

The main function of BAT is thermogenesis, which requires the action of UCP1. UCP1 increases the permeability of the inner mitochondrial membrane to reduce mitochondrial membrane potential and thereby uncouple ATP generation from the respiratory chain leading to heat generation (reviewed in reference 58). Interestingly, we found reduced UCP1 protein and mRNA in the BAT of AdHIF2KO mice compared to those in littermate HIF2α-proficient mice under obese (Fig. 9A, B, and F, data from mice fed an HFD for 24 weeks; data from mice fed an HFD for 16 weeks are not shown) but not lean conditions (data not shown). These data suggested that HIF2α-mediated regulation of UCP1 is operative only during HFD-induced metabolic deterioration of BAT. In contrast, other BAT markers were not changed between obese control and AdHIF2KO mice (Fig. 9C).

Because of the essential role of UCP1 in heat generation, we performed a cold resistance test in HFD-fed AdHIF2KO and control littermate mice. Obese AdHIF2KO mice were unable to maintain adequate body temperature, in contrast to HFD-fed control mice (Fig. 9D and E). Thus, not only is HIF2α expression induced in mice subjected to cold exposure (25) but also adipocyte HIF2α contributes to cold adaptation. Furthermore, gene expression analysis revealed a significant reduction of UCP1 expression in the BAT of obese AdHIF2KO mice compared to that in littermate control mice under both cold and room temperature conditions (Fig. 9F). Additionally, reduced expression of *Vegf-a* was observed in the BAT of obese AdHIF2KO mice under both cold and room temperature conditions (Fig. 9F) compared to that in obese HIF2α-proficient mice. Together, these findings demonstrate that adipocyte HIF2α deficiency promotes obesity-related BAT dysfunction.

### BAT dysfunction and enhanced AT inflammation at early stages of obesity in AdHIF2KO mice.

**FIG 10:**
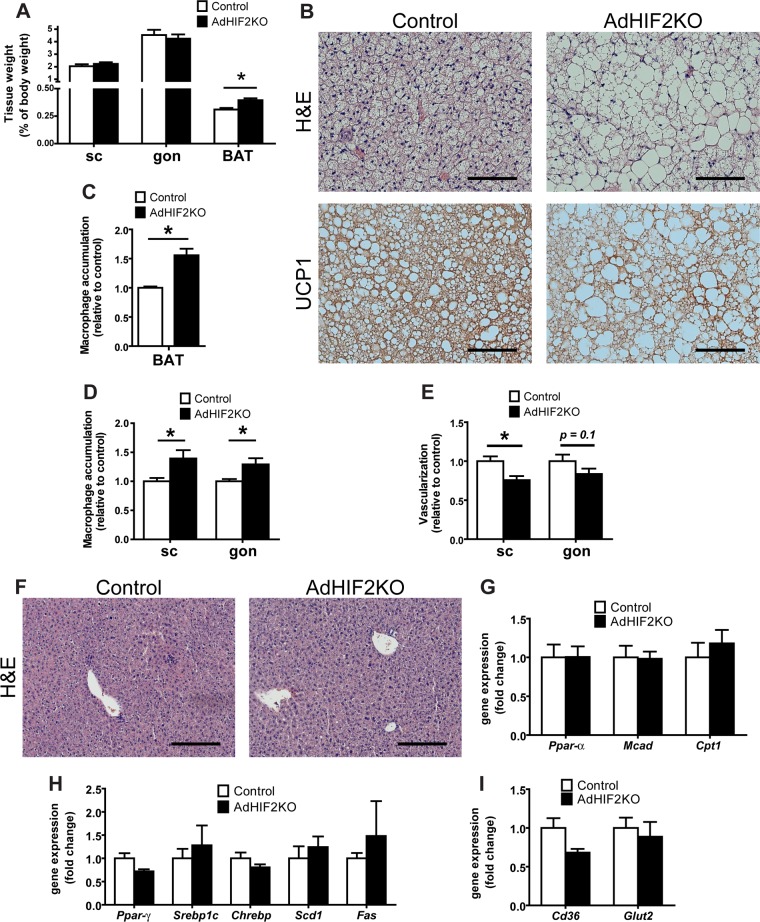
Metabolic dysregulation of WAT and BAT in AdHIF2KO mice in early stages of diet-induced obesity. Control and AdHIF2KO mice were fed with an HFD for 4 weeks. (A) Subcutaneous (sc) and gonadal (gon) WAT and BAT were weighed. The tissue weights are presented as percentages of total body weight. (B) Representative images from H&E staining and UCP1 immunohistochemistry in BAT from control and AdHIF2KO mice. Scale bars are 100 μm. (C) Quantification of immunohistochemistry for F4/80 in BAT from both genotypes. Macrophage accumulation of control mice was set as 1. (D) Quantification of immunohistochemistry for F4/80 in scWAT and gonWAT from control and AdHIF2KO mice. Macrophage accumulation of control mice was set as 1. (E) Vessels were stained for isolectin B4 in whole mounts of scWAT and gonWAT from control and AdHIF2KO mice and quantified. Vascularization of control mice was set as 1. (F) Representative images from H&E staining in livers from control and AdHIF2KO. Scale bars are 200 μm. (G to I) Gene expression analysis for lipolytic (G) and lipogenic (H) markers as well as transporters for FFA (*Cd36*) and glucose (*Glut2*) (I) in livers from obese control and AdHIF2KO mice. Gene expression of control mice was set as 1. Data in panels A, C to E, and G to I are expressed as means ± SEMs (*n =* 6 mice per group). *, *P* < 0.05.

In order to understand the mechanisms underlying the metabolic alterations seen in AdHIF2KO mice, we analyzed mice fed with an HFD for only 4 weeks, hence, before body mass differences are established between AdHIF2KO and control mice. In contrast to longer feedings (16 to 24 weeks [Fig. 2B and data not shown]), AdHIF2KO mice fed for only 4 weeks did not show any difference in WAT weights, while BAT weight was already significantly elevated (Fig. 10A). Moreover, brown adipocytes were enlarged in AdHIF2KO mice already at early stages of obesity (Fig. 10B). In addition, UCP1 expression was reduced and macrophages were more abundant in the BAT from AdHIF2KO mice after only 4 weeks of diet (Fig. 10B and C). Despite unchanged tissue mass, scWAT and gonWAT from AdHIF2KO mice displayed enhanced accumulation of macrophages, associated with reduced vascularization, indicating WAT dysfunction due to HIF2α deficiency in adipocytes in early stages of obesity (Fig. 10D and E). In contrast, lipid accumulation in the liver, as well as markers for lipolysis, lipogenesis, and FA uptake, were not altered in AdHIF2KO mice compared to those in control mice after 4 weeks of HFD feeding (Fig. 10F to I). Together, these data indicate that enhanced inflammation and dysfunction especially of BAT are already present at early stages of diet-induced obesity due to adipocyte HIF2α deficiency and are likely the primary events leading to metabolic dysregulation in these mice.

### VEGF rescues BAT dysfunction in obese AdHIF2KO mice.

**FIG 11:**
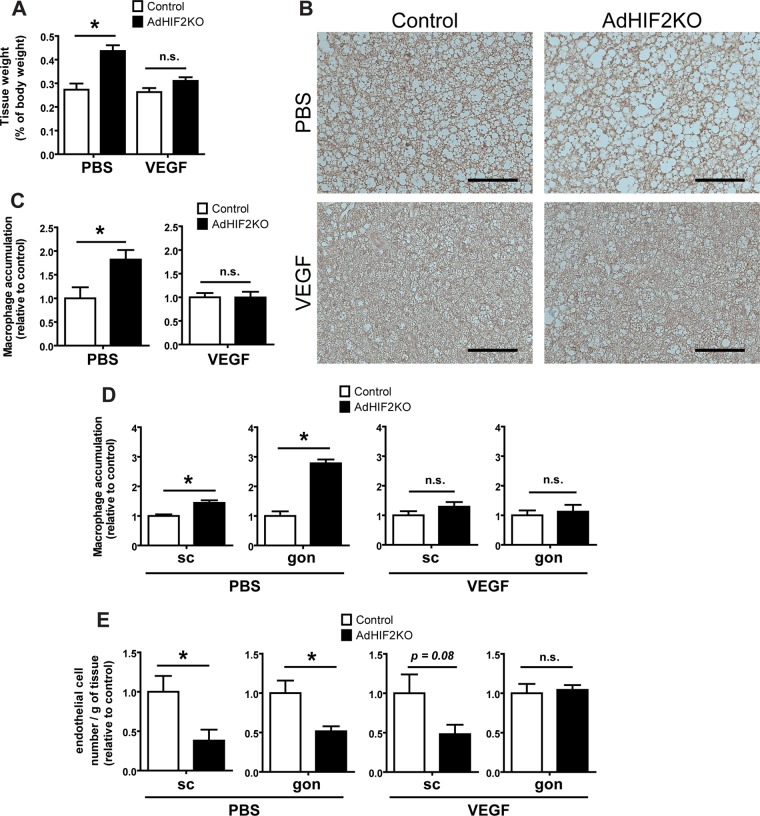
VEGF administration reverses metabolic dysregulation of the BAT associated with adipocyte HIF2α deficiency. Control and AdHIF2KO were fed with an HFD for a total of 8 weeks; for the last 3 weeks of the experiment, mini-osmotic pumps were implanted subcutaneously for administration of murine VEGF or PBS as a control. (A) BAT weight after PBS or VEGF treatment. The tissue weight is presented as percentage of total body weight. (B) Representative images for UCP1 staining in BAT from control and AdHIF2KO mice. Scale bars are 100 μm. (C) Quantification of immunohistochemistry for F4/80 in BAT from control and AdHIF2KO mice treated with either PBS or VEGF. Macrophage accumulation of control mice was set as 1. (D) Quantification of immunohistochemistry for F4/80 in subcutaneous (sc) and gonadal (gon) WAT from control and AdHIF2KO mice treated with either PBS or VEGF. Macrophage accumulation of control mice was set as 1 in each case. (E) Flow cytometry analysis for CD31^+^ CD45^−^ cells was performed to analyze absolute endothelial cell numbers. The absolute endothelial cell number per gram of tissue was quantified. Data are shown relative to those for control mice; data for control mice were set as 1 in each case. Data in panels A and C to E are expressed as means ± SEMs (*n =* 3 to 6 mice per group). *, *P* < 0.05.

Mice lacking HIF2α in adipocytes showed BAT dysfunction already at early stages of obesity (Fig. 10A to C), accompanied by reduced VEGF expression in the BAT (Fig. 8H). We therefore addressed next whether the impaired levels of this major proangiogenic factor could contribute to elevated BAT dysfunction and inflammation. To this end, obese control and AdHIF2KO mice were subjected to VEGF administration via mini-osmotic pumps or PBS as a control treatment. VEGF treatment reversed the enhanced weight of obese BAT due to adipocyte HIF2α deficiency (Fig. 11A). Consistently, VEGF treatment reversed the HIF2α deficiency-associated reduced UCP1 expression of BAT and elevated macrophage accumulation in BAT (Fig. 11B and C). In other words the BAT dysfunction in adipocyte HIF2α deficiency was reversed, at least partially, by VEGF administration. Additionally, VEGF administration efficiently reversed the exacerbated macrophage accumulation and the reduced vascularization in the WAT of obese AdHIF2KO mice (Fig. 11D and E). Together, these data demonstrate that the reduced levels of VEGF resulting from adipocyte HIF2α deficiency contribute to BAT inflammation and dysfunction (reduced UCP1 expression) in obese AdHIF2KO mice.

## DISCUSSION

In this study, we identified HIF2α in white and brown adipocytes as an important factor counteracting the maladaptation of WAT and BAT to obesity. Especially, this work demonstrates for the first time that adipocyte HIF2α contributes to the regulation of the adaptation of BAT to obesity. Accordingly, obese AdHIF2KO mice displayed BAT dysfunction, such as increased BAT mass, enlarged brown adipocytes, and decreased UCP1 gene and protein expression.

Metabolic dysregulation of BAT in obese AdHIF2KO mice was linked to a dysfunctional thermogenic response of these mice to cold exposure. In particular, obese AdHIF2KO mice exposed to cold failed to sustain their body temperature. These data are in keeping with the previous observation that cold exposure induced HIF2α expression in the AT (25). In fact, BAT hypoxia not only is present in the course of obesity (3) but also is one of the earliest events during cold challenge, likely resulting from the excessive demand of oxygen that is required for UCP1 activity and heat generation (25).

It is noteworthy that we found that the expression of the major thermogenic factor UCP1 was altered in adipocyte HIF2α deficiency. In contrast, a recent report did not show alterations in UCP1 expression in the BAT of adipocyte-specific HIF1α-deficient mice compared to adipocyte HIF1α-proficient mice in obesity (6). Although the underlying mechanisms of UCP1 regulation by adipocyte HIF2α are not known and merit future investigations, our findings are consistent with previous work showing that mice with adipocyte-specific deficiency of PHD2, which is a negative regulator of HIF2α, showed enhanced UCP1 expression in obese BAT (15). VEGF has been previously shown to promote UCP1 expression (3, 11, 57). The fact that VEGF treatment rescued the reduced UCP1 expression in HIF2α-deficient brown adipocytes suggests that the regulation of UCP1 by HIF2α may involve VEGF. Moreover, UCP1 expression was not affected in lean AdHIF2KO mice compared to that in control mice, indicating that the HIF2α-mediated regulation on UCP1 may be operative in adaptive rather than constitutive BAT responses, such as under chronic excessive lipid consumption characteristic of obesity or under acute cold exposure. Taken together, our data demonstrate that adipocyte HIF2α is one of the factors that contribute to BAT adaptation to obesity.

Furthermore, deletion of adipocyte HIF2α resulted in enhanced WAT dysfunction only in the obese and not in the lean state, as indicated by reduced WAT angiogenesis, enhanced WAT inflammation, increased WAT fibrosis, and adipocyte death. Consistent with the reduced expression of lipases and of genes involved in lipid oxidation, with the reduced *ex vivo* lipolysis and the reduced lipid tolerance in adipocyte HIF2α deficiency, we found enhanced blood cholesterol levels, reduced serum free fatty acids, and ectopic fat accumulation in the liver. AdHIF2KO mice subjected to a short HFD feeding (4 weeks) displayed BAT dysfunction and enhanced AT inflammation compared to those in control mice, whereas liver lipid accumulation was not affected at the early stage of obesity. A recent study briefly showed that lack of HIF2α in adipocytes enhanced body weight and gonWAT inflammation and promoted insulin resistance (6) without addressing WAT angiogenesis or assessing further metabolic organs apart from the gonWAT, such as the scWAT and the liver, and more importantly without reporting the role of adipocyte HIF2α in BAT, as we have thoroughly done here. We provide here crucial detailed insights with regard to WAT and BAT dysfunction in obese AdHIF2KO mice.

A recent report has shown that heterozygous HIF2α+/− mice are prone to insulin resistance and AT inflammation in obesity (17); these authors attributed their findings to the absence of HIF2α from macrophages because clodronate-mediated macrophage depletion improved glucose intolerance in HIF2α+/− mice. However, the more specific strategy employed in this study (myeloid cell-specific HIF2α deficiency) did not reveal a role of macrophage HIF2α in obesity-related WAT inflammation and metabolic dysregulation. It is likely that the phenotype of heterozygous HIF2α+/− mice in obesity resulted from the partial deficiency of adipocyte HIF2α. Macrophage-mediated WAT inflammation is a common downstream event of several pathways leading to WAT dysfunction, and several examples exist showing that amelioration of WAT inflammation improves obesity-related insulin resistance (59). Thus, it is quite possible that obesity-related metabolic dysregulation initiated by WAT dysfunction due to adipocyte-specific HIF2α deficiency could be improved by manipulating a downstream effector, e.g., by macrophage ablation. Together, our data clearly demonstrate that adipocyte but not endothelial or myeloid HIF2α orchestrates the intimate cross talk between adipocytes, macrophages, and the endothelium within the WAT.

Although Fabp4-Cre transgenic mice have been extensively used to achieve adipocyte-specific deletion, some degree of recombination in other cells and tissues, such as macrophages or endothelial cells, has been reported (53). In the present work, we did not find significant HIF2α deletion in the liver, skeletal muscle, heart, hypothalamus, or bone marrow macrophages of AdHIF2KO mice. This finding, together with the absence of any phenotype in the myeloid cell- and endothelium-specific HIF2α-deficient mice in diet-induced obesity, allow us to conclude that the observed phenotypes in AdHIF2KO mice essentially derive from the deletion of HIF2α in adipocytes. Our results show that adipocyte but not myeloid or endothelial HIF2α is responsible for regulating the angiogenic response within the WAT in obesity and for reducing obesity-related WAT inflammation and metabolic dysregulation.

Our findings support and extend a previous report demonstrating that vascular rarefaction by adipocyte-specific deletion of VEGF promotes BAT dysfunction and whitening (3). Although previous reports have shown that hypoxia in obesity induces HIFs (2, 6, 12) and have illustrated the importance of proangiogenic responses in WAT and BAT adaptation to obesity (3, 8, 9, 11, 57), the involvement of HIF1α or HIF2α in this process was not clarified. In fact, several lines of evidence have suggested that HIF1α does not participate in regulating *Vegf-a* expression or in inducing angiogenesis in WAT or BAT. On the other hand, adipocytes lacking HIF1β, and thereby HIF1α and HIF2α signaling, showed reduced VEGF-A expression (21). Additionally, transgenic overexpression of HIF1α in adipocytes did not enhance VEGF-A expression (12), and mice with deletion of adipocyte-specific HIF1α had no alterations in endothelial cell numbers compared to those in HIF1α-proficient mice (6). Similarly, HIF1α overexpression in brown adipocytes did not enhance *Vegf-a* expression (3). These findings on the role of HIF1β and HIF1α, together with our present findings of reduced VEGF-A levels, endothelial cell numbers, and vascularity of WAT and BAT in obese adipocyte-specific HIF2α-deficient mice, unequivocally underline the primacy of adipocyte HIF2α as the major HIF isoform orchestrating the angiogenic response in the WAT and BAT in obesity. The clarification of the distinct actions of HIF1α and HIF2α in adipocytes adds yet another example to the variable, nonredundant, and often opposite functions these two transcription factors have in several biological processes (1).

In conclusion, we demonstrate that adipocyte HIF2α regulates angiogenesis in obese WAT and BAT. Furthermore, adipocyte HIF2α is integral to the thermogenic response of BAT in obesity by regulating UCP1 expression. Through these complementary mechanisms, adipocyte HIF2α counteracts BAT dysfunction, AT inflammation, and metabolic dysregulation and insulin resistance in obesity.
